# Stabilization of Transaminases in Aqueous-Organic Media by Pyridoxal-5’-phosphate: A Case Study of Transaminase from Desulfomonile tiedjei

**DOI:** 10.32607/actanaturae.27820

**Published:** 2026

**Authors:** I. V. Rudina, A. K. Bakunova, V. O. Popov, E. Yu. Bezsudnova

**Affiliations:** A.N. Bach Institute of Biochemistry, Research Center of Biotechnology of the Russian Academy of Sciences, Moscow, 119071 Russia; Department of Biology, M. V. Lomonosov Moscow State University, Moscow, 119991 Russia

**Keywords:** transaminases, enzyme catalysis, stability, organic solvents

## Abstract

Pyridoxal-5’-phosphate (PLP)-dependent transaminases are highly efficient biocatalysts for the stereoselective synthesis of chiral amines, which are key building blocks in pharmaceuticals and chemical manufacturing. Fundamental research on enzymatic transamination includes the classical works of Alexander Braunstein, who discovered the transamination reaction; David Metzler, who studied the spectral properties of PLP-dependent enzymes; Esmond Snell, who investigated the kinetics of PLP-dependent enzymes; as well as studies by other Russian and international researchers. Despite extensive studies on PLP-dependent transaminases, their practical application remains limited. In addition to the unfavorable equilibrium of the transamination reaction and the narrow substrate specificity of transaminases, their stability under manufacturing conditions is a major constraint. Transaminase stability encompasses not only the structural integrity of the protein globule, but also the enzyme’s ability to retain the PLP cofactor. PLP dissociation leads to enzyme inactivation and termination of the reaction. Modern biocatalytic processes are predominantly designed for aqueous–organic media to increase the solubility of hydrophobic substrates to hundreds of grams per liter. Under these conditions, the stability of transaminases, as with other enzymes, decreases. In the context of these challenges, this work investigates the efficiency of PLP binding as a factor in stabilizing the active holoenzyme of the transaminase from *Desulfomonile tiedjei *in various aqueous–organic media. The study analyzes the transaminase stability and catalytic activity in the presence of methanol, DMSO, and Cyrene (up to 20% *v*/*v*), both in incubation mode and under reaction conditions. Particular attention is paid to the analysis of the effect of the amino acid substitution T199Q in the cofactor-binding region on the enzyme’s resistance to organic solvents. The present study contributes to addressing the practical problem of stabilizing transaminases in aqueous–organic media. The results also deepen our understanding of the molecular basis of the stability of PLP-dependent enzymes.

## INTRODUCTION

Pyridoxal-5′-phosphate (PLP)-dependent transaminases [EC 2.6.1.X] catalyze the stereoselective transfer of an amino group from an amino acid/amine to an α-keto acid/ketone, yielding a new amino acid/ amine and a new α-keto acid/ketone [1, 2, 3]. Enzymatic transamination is a double-displacement reaction involving transient transfer of the amino group to the PLP cofactor during deamination of the amino acid substrate, resulting in the formation of pyridoxamine- 5′-phosphate (PMP). PMP then serves as an amino group donor in the second half-reaction: amination of the second keto acid substrate. Transaminases have been studied for more than 80 years. The fundamen tal studies in this area were conducted by Alexander Braunstein, who discovered the transamination reaction; David Metzler, who studied the spectral properties of pyridoxal enzymes and the equilibrium of individual steps of the transamination reaction; Esmond Snell, who investigated the kinetics of PLP-dependent enzymes; as well as other Russian and international researchers [3, 4, 5, 6]. The use of transaminases for stereoselective amination of keto compounds on an industrial scale was first proposed in 2010 [7]. To date, a number of biotechnological processes using transaminases have already been established for industrial application and new methods continue to be proposed [8, 9, 10, 11, 12, 13, 14]. To create biocatalysts based on transaminases, it is important to address challenges such as increasing their specificity towards the target substrate, shifting the equilibrium of the transamination reaction towards product formation, and stabilizing the enzyme under reaction conditions [10, 14, 15]. The stability issue involves not only thermal stability, but also the maintenance of the active conformation of the biocatalyst under operational conditions [16, 17]. In the case of transaminases, this primarily involves stabilization of the holoenzyme in aqueous–organic media, since an organic solvent (up to 50% *v*/*v*) is added to increase the solubility of the keto substrate [7, 10]. In addition, cofactor dissociation from the active site of the transaminase may occur, leading to enzyme inactivation [17, 18, 19]. The simplest and most effective way to enhance holoenzyme stability is to add an excess of the PLP cofactor into the reaction medium [7, 10, 19]. However, this approach does not fully address the issue of cofactor release in the form of pyridoxamine-5′-phosphate (PMP), which is an intermediate cofactor form generated after amino group transfer from the amino acid substrate [1]. Under reaction conditions, stabilization can be achieved either by increasing the concentration of the second substrate (keto compound, or the amino group acceptor) or by introducing amino acid substitutions in the cofactor- binding region [17, 19].

**Fig. 1:**
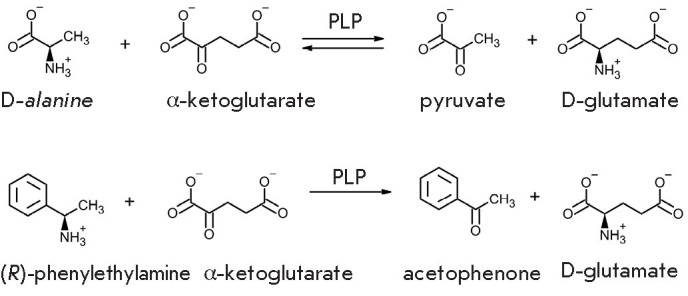
Schemes of transamination reactions

**Fig. 2:**
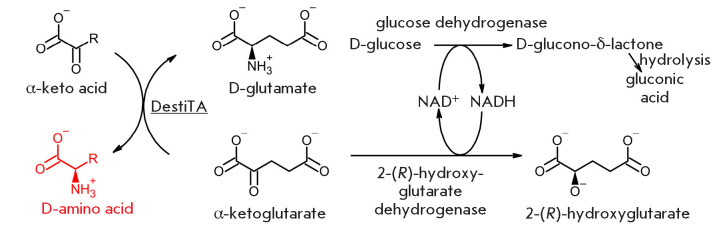
Three-enzyme system for the synthesis of D-amino acids

We have previously characterized the transaminase DestiTA from the Gram-negative bacterium* Desulfomonile tiedjei*. DestiTA efficiently catalyzes the transfer of an amino group from both D-amino acids and *(R)*-phenylethylamine to α-ketoglutarate and other keto acids (*Fig. 1*) [20]. DestiTA also functions effectively in a three-enzyme system for the stereoselective amination of α-keto acids, producing D-amino acids with up to 99% yield and an enantiomeric excess exceeding 99% (*Fig. 2*) [20].

**Fig. 3:**
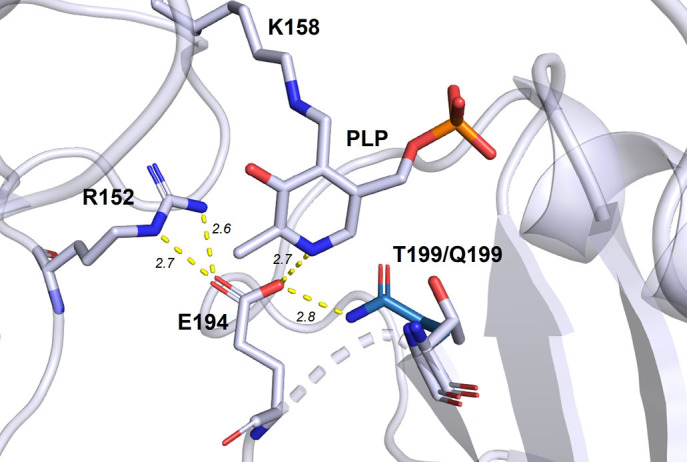
Cofactor binding in the DestiTA active site. The amino acid substitution T199Q that potentially leads to the formation of a hydrogen bond Q199/NE2–E194/OE2 is shown. Distances are presented in Angstroms.

At the same time, instability of the DestiTA holoenzyme has been observed. In other words, the apoenzyme predominates in the reaction buffer and efficient enzyme functioning requires the addition of excess PLP into the reaction mixture. To stabilize the holoenzyme, we substituted individual amino acid residues in the second coordination sphere of the PLPbinding site in the DestiTA active site. The T199Q substitution was found to be the most effective, since it enabled the formation of a hydrogen bond between Gln199 and the conserved Glu194, which coordinates the N1 atom of the PLP molecule (*Fig. 3*). The T199Q substitution resulted in a five-fold decrease in the holoenzyme dissociation constant (from 11 ± 1 μM for the wild-type (WT) enzyme to 2.2 ± 0.4 μM for the T199Q variant) and a slight increase in the *Vmax *of the transamination reaction between D-alanine and α-ketoglutarate [20]. The thermal denaturation midpoint (*T*0.5) increased by 1°C for the T199Q holoen zyme and decreased by 5°C for the apoenzyme. We further investigated the effect of the T199Q substitution on DestiTA functioning and analyzed the stability and catalytic efficiency of the holoenzyme in aqueous–organic solutions.

## EXPERIMENTAL

**Preparation of recombinant DestiTA and its T199Q variant**

Purified, active recombinant WT DestiTA and the T199Q variant were obtained as previously described [20]. The purity and homogeneity of the enzymes were assessed by denaturing polyacrylamide gel electrophoresis. Protein concentration was determined spectrophotometrically by measuring the absorbance at 280 nm.

**Standard reaction**

The standard transamination reaction between D-alanine and α-ketoglutarate was performed in 50 mM K-phosphate buffer (pH 8.0), at 40°C, D-alanine and α-ketoglutarate concentrations were 25 and 10 mM, respectively, in the presence of 30 μM PLP, 330 μM NADH, and 2 U/mL rabbit lactate dehydrogenase (LDH) (Sigma, USA). The reaction was initiated by adding a DestiTA aliquot (0.004 mg/mL) after equilibrating the reaction mixture to 40°C. The reaction progress was monitored using a coupled enzymatic reaction catalyzed by LDH, in which pyruvate formed during the transamination reaction serves as a substrate. LDH catalyzes the conversion of pyruvate to lactate in the presence of NADH. NADH oxidation was monitored at 340 nm (εNADH = 6.22 mM-1cm-1). Measurements were performed in a quartz cuvette with a 0.5 cm optical pathlength using an Evolution 300 UV–Vis spectrophotometer (Thermo Fisher Scientific, USA). DestiTA specific activity was expressed as U/mg of the enzyme, where 1 U corresponds to the conversion of 1 μmol of the substrate per minute.

**Determination of C50(PLP) for WT DestiTA and the T199Q variant**

PLP binding was studied by kinetic analysis in a reaction mixture with LDH similar to that described above, but with substrate concentrations of 100 mM D-alanine and 10 mM α-ketoglutarate. PLP concentrations ranged from 0 to 30 μM. The reaction was initiated by adding the apo form of DestiTA to a final concentration of 0.01 mg/mL (0.3 μM).

**Determination of the kinetic parameters of the half-reaction between DestiTA and D-valine**

The interaction of the DestiTA holoenzyme (PLP form) (0.72 mg/mL (20 μM)) with D-valine (0–0 mM) was analyzed spectrophotometrically using a SPECTROstar Omega microplate reader. The half-reaction was monitored at 410 nm in 50 mM K-phosphate buffer (pH 8.0) at 40°C.

Under substrate excess conditions, the half-reaction was assumed to follow first-order kinetics. The observed half-reaction rate constant *k*_obs_ was determined by fitting the absorbance data over time using equation (1):

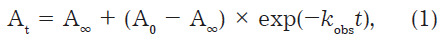



where At is the absorbance at time *t*, A0 is the initial absorbance, and A∞ is the final absorbance.

The kinetic parameters of the half-reaction were calculated using equation (2):

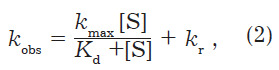



where [S] is the substrate concentration, *k*_max_ is the maximum half-reaction rate constant, *K*_d_ is the enzyme- substrate complex dissociation constant, *k*_r_ is the rate constant showing the reverse reaction contribution, and *k*_max_/*K*_d_ is the specificity constant.

**Effect of organic solvents on enzyme specific activity in the transamination reaction**

The effect of organic solvents on DestiTA activity was analyzed using the standard transamination reaction between D-alanine and α-ketoglutarate. For this, 10–20% (*v/v*) DMSO, methanol, or Cyrene was added into the reaction mixture.

Due to the high optical density of Cyrene at 340 nm and the denaturation of LDH in the presence of 20% (*v/v*) methanol, aliquots were taken from the reaction mixture at set time intervals to assay enzymatic activity. The reaction was stopped by heating at 98°C for 5 min. The concentration of pyruvate formed in each aliquot was then determined using LDH at 25°C in 50 mM K-phosphate buffer (pH 8.0) from a linear calibration curve of LDH activity as a function of pyruvate concentration.

**Determination of the observed rate constant of dissociation of the DestiTA holoenzyme during incubation in buffer and under reaction conditions in the presence of organic solvents**

The rate constant of dissociation of the DestiTA holoenzyme was determined spectrophotometrically by monitoring the absorbance decay at 430 nm, which corresponds to the loss of the holo form; the extinction coefficient was 0.2 mL×mg-1cm-1. For this, 0.2–0.6 mg/mL DestiTA was incubated in 50 mM K-phosphate buffer, either without substrates or in the presence of 100 mM D-alanine and 10 mM α-ketoglutarate, at 40°C. To evaluate the effect of organic solvents, 10–0% (*v/v*) DMSO, methanol, or Cyrene was added into reaction mixtures. Precipitate formation was monitored at λ = 500–550 nm. The observed rate constant of holoenzyme dissociation was calculated using equation (3):

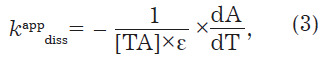



where *k*_app_ diss is the rate constant of holoenzyme dissociation, [TA] is the transaminase concentration, ε is the extinction coefficient of the holoenzyme at 430 nm, and dA/dT is the experimental curve slope. The Origin 8.0 software (OriginLab, USA) was used to process the experimental curves

**Determination of the thermal denaturation midpoint for WT DestiTA and the T199Q variant**

Differential scanning fluorimetry was used to determine the thermal denaturation midpoint (*T*0.5) of the apoenzyme and holoenzyme of DestiTA in 50 mM K-phosphate buffer, pH 8.0. The apoenzyme was obtained by incubating the enzyme with 2 mM phenylhydrazine for 20 min at room temperature, followed by buffer exchange using a desalting column (Cytiva, USA). The holoenzyme was obtained by incubating the enzyme with 300 μM PLP for 1 h at room temperature. The holoenzymes at a final concentration of 0.07 mg/mL (2 μM) were mixed with 25×ProteOrange Protein Gel Stain (Lumiprobe, USA). The effect of organic solvents was analyzed by adding 10–0% (*v/v*) methanol, DMSO, or Cyrene into the samples. Measurements were conducted at 515–530 nm excitation and 560–580 nm emission wavelengths using a CFX96 RT-PCR system (Bio-Rad, USA) with temperature increments of 0.2°C, followed by sample equilibration for 10 s over a temperature range of 25–0°C. Three experimental curves were obtained for each sample. Data were processed using the CFX Manager software (Bio-Rad) and further analyzed in OriginPro 8.0. The thermal denaturation midpoint (*T*0.5) was determined as the maximum of the first derivative of the fluorescence-temperature curve.

**Analysis of the operational stability of WT DestiTA and T199Q in the presence of organic solvents**

The operational stability of DestiTA was analyzed by incubating the enzyme at a concentration of 0.2 mg/mL in 50 mM K-phosphate buffer, pH 8.0, supplemented with 50 mM D-glutamate, 50 mM 3-methyl-2-oxobutyrate, 300 μM PLP, and either 10–0% (*v/v*) DMSO or 10% (*v/v*) methanol at 40°C. To assess DestiTA activity, aliquots were collected immediately after preparation and after 1, 2, and 5 days of incubation. Enzyme activity was measured using the standard transamination reaction with D-alanine and α-ketoglutarate.

## RESULTS AND DISCUSSION

**Stability of the DestiTA holoenzyme under reaction conditions: the effect of the T199Q substitution**

**Fig. 4:**
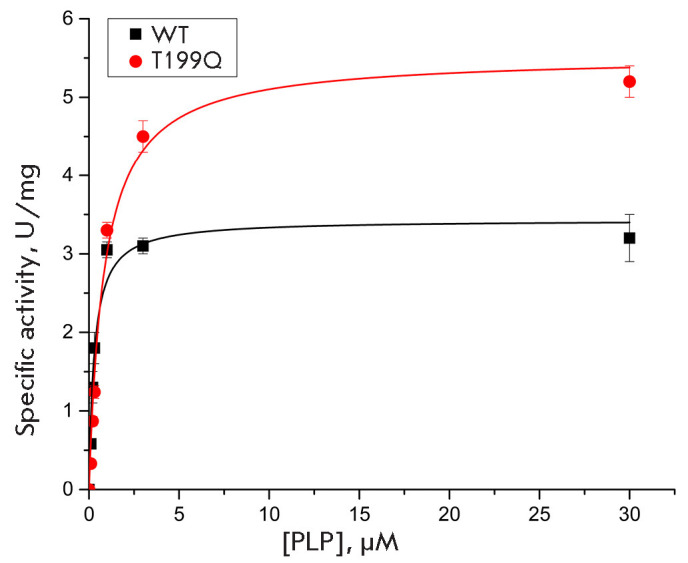
Dependence of the specific activities of WT DestiTA and T199Q on the PLP concentration in the transamination reaction between 100 mM D-alanine and 10 mM α-ketoglutarate (saturating concentrations) in 50 mM K-phosphate buffer (pH 8.0) at 40°C

We have previously compared the properties of WT DestiTA and the T199Q variant and observed a decrease in the dissociation constant of the DestiTA holoenzyme upon introduction of the T199Q substitution; this, based on modeling results, is associated with the formation of an additional hydrogen bond between Gln199 and Glu194 in the cofactor-binding region [20]. In this work, we analyzed the stability of the WT and T199Q holoenzymes under reaction conditions. For this, we assessed the efficiency of holoenzyme formation from the DestiTA apoenzyme and free PLP under reaction conditions using the C50(PLP) parameter (*Fig. 4*). The obtained C50 values were 0.29 ± 0.07 and 0.8 ± 0.1 μM for WT DestiTA and T199Q, respectively, indication that the T199Q substitution destabilizes the holoenzyme under reaction conditions. To further clarify this observation, we analyzed substrate binding to the wild-type and variant enzymes in the first half-reaction of deamination [1] in the absence of the second substrate. The kinetic parameters of the half-reaction of the WT holoenzyme and T199Q variant with D-valine were *k*_max_ = 0.068 ± 0.004 s-1 and *K*_D_ = 0.5 ± 0.1 mM, and *k*_max_ = 0.019 ± 0.001 s-1 and *K*_D_ = 10 ± 2 mM, respectively. Apparently, the substitution at the PLP cofactor-binding site reduced the substrate-binding affinity, which affected C_50_(PLP) and the kinetic stability of DestiTA.

**DestiTA activity in aqueous-organic media**

To study DestiTA stability in aqueous-organic media, DMSO and methanol were selected as solvents commonly used in biocatalytic processes involving transaminases [8, 9, 10, 10]. This choice was based on the differences in the key physicochemical properties of DMSO and methanol: hydrophobicity (logP = −1.35 and −0.74 for DMSO and methanol, respectively), the dipole moment (3.96 and 1.70 D for DMSO and methanol, respectively), and the interaction with water molecules. Methanol acts as a hydrogen bond donor, whereas DMSO is a hydrogen bond acceptor. This allows for a comprehensive assessment of the effects of different types of solvents on enzyme stability. We also tested the Cyrene solvent (logP = −1.52; 0.93 D; hydrogen bond acceptor), which is promoted as a biodegradable, non-mutagenic, and non-toxic alternative to traditional dipolar aprotic solvents such as DMSO, N,N-dimethylformamide (DMF), dioxane, and tetrahydrofuran (THF) [21, 22]. The effect of organic solvents on enzyme activity was assessed by measuring the specific activity of DestiTA in the transamination reaction with D-alanine and α-ketoglutarate as substrates (*Fig. 1*) in 50 mM K-phosphate buffer (pH 8.0) at 40°C (*Table 1*). A positive effect of the T199Q substitution on DestiTA activity was observed in DMSO, whereas the variant exhibited lower activity in 10−20% (*v/v*) Cyrene.

**Table 1:** Specific activity of WT DestiTA and its variant T199Q in the transamination reaction between D-alanine and α-ketoglutarate (unsaturating concentrations) in a 50 mM K-phosphate buffer (pH 8.0) at 40°C

Organic solvent, %		Specific activity, U/mg	
		WT	T199Q
no solvent		3.4 ± 0.2	2.8 ± 0.3
Cyrene	10	3.7 ± 0.1	2.9 ± 0.2
	20	2.3 ± 0.3	2.0 ± 0.2
DMSO	10	2.7 ± 0.1	2.8 ± 0.1
	20	2.1 ± 0.2	3.0 ± 0.1
Cyrene	10	2.4 ± 0.3	1.8 ± 0.2
	20	2.0 ± 0.2	No activity

**Thermodynamic and kinetic stability of DestiTA in water-organic solvent media: effect of the T199Q substitution**

**Table 2:** Effect of organic solvents on the thermal denaturation midpoint (T0.5) of WT DestiTA and T199Q in 50 mM K-phosphate buffer (pH 8.0) with the addition of organic solvents

Organic solvent, %	T_0.5_, °C	
	WT	T199Q
no solvent	64.4 ± 0.2	65.7 ± 0.1
Methanol, 10	59.9 ± 0.3	61.2 ± 0.4
Methanol, 20	49.5 ± 0.6	53.0 ± 0.2
DMSO, 20	57.7 ± 0.5	58.1 ± 0.2
Cyrene, 20	40.6 ± 0.8	42.7 ± 0.8

The thermodynamic stability of the holoenzyme increased upon introduction of the T199Q substitution not only in the buffer alone [20] but also in buffer supplemented with organic solvents (*Table 2*). To analyze the stability of DestiTA, the enzyme’s thermal denaturation midpoint (*T*0.5) was determined by differential scanning fluorimetry. Substitution T199Q appeared to stabilize the enzyme, whereas cyrene was the most destructive for DestiTA, decreasing* T*0.5 by more than 20°C. The effects of methanol and DMSO were less pronounced. The observed effects were different than the solvent’s effects on activity (*Table 1*). Apparently, these effects are determined by the ability of solvents to form hydrogen bonds, as well as the hydrophobicity, the dipole mo ment (µ), etc., to varying degrees depending on the experimental conditions.

**Table 3:** Holoenzyme dissociation under reaction conditions (saturating substrate concentrations) with the addition of organic solvents in 50 mM K-phosphate buffer (pH 8.0) at 40°C

Organic solvent, %		k^app^_diss_ × 10^-3^, min^-1^	
		WT	T199Q
no solvent		12.8 ± 0.3	100 ± 20
Methanol	10	27 ± 1	280 ± 15
	20	precipitate	precipitate
DMSO	10	12.7 ± 0.4	110 ± 15
	20	7.1 ± 0.4	120 ± 10
Cyrene	10	precipitate	precipitate

**Fig. 5:**
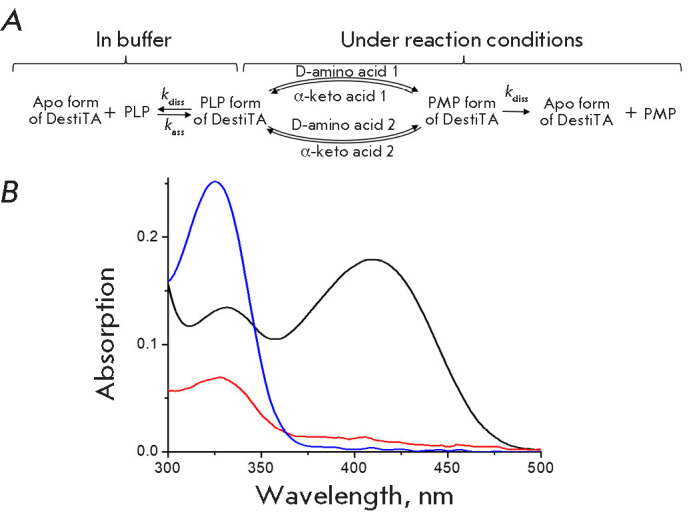
Scheme of DestiTA inactivation in buffer and under reaction conditions (*A*). Absorption spectra of the PLP (black) and PMP (blue) forms of the holoenzyme and apoenzyme (red) (*B*). The spectra are normalized to absorbance at 280 nm

The kinetic stability of WT DestiTA and T199Q was evaluated via the rate of cofactor dissociation from the active site, leading to enzyme inactivation. The dissociation was assessed by monitoring changes in the holoenzyme absorption spectrum (*Fig. 5*). The experiment was performed both in buffer and under reaction conditions; i.e., in the presence of the two substrates D-alanine and α-ketoglutarate, the latter implying the presence of the cofactor in both the PLP and intermediate PMP forms. The observed holoenzyme dissociation rate constant (*k*app diss) in 50 mM K-phosphate buffer (pH 8.0) in the absence of substrates was comparable for WT DestiTA and T199Q and equal to (12.2 ± 0.4) × 10-3 and (20 ± 4) × 10-3 min-1 at 40°C, respectively. Under reaction conditions, the observed T199Q rate constant of dissociation (*k*app diss) was almost an order of magnitude higher, indicating its lower kinetic stability compared to the WT enzyme (*Table 3*).

Whereas the thermal denaturation midpoint (*T*0.5) can be considered with caution as an indicator of protein thermodynamic stability (this is true only for reversible unfolding of the protein; DestiTA undergoes aggregation upon heating), the holoenzyme dissociation constant represents a more objective measure of holoenzyme thermodynamic stability [20]. Evidently, the holoenzyme variant is more stable than the WT holoenzyme. However, its kinetic stability, as reflected by *k*app diss, which determines the rate of inactivation, is lower for T199Q. It is possible that changes in kinetic parameters due to the substitution in the active holoenzyme (see section above) also affect the kinetic stability. In the case of DestiTA, the WT enzyme appears to be more stable than T199Q, particularly under reaction conditions. This may be associated with the reduced stability of the T199Q apoenzyme, which accumulates during catalysis, especially in the absence of free PLP [19].

As for the solvent effects, DMSO stabilizes the WT holoenzyme but has little effect on the T199Q variant under reaction conditions in the presence of substrates. The WT enzyme’s specific activity decreases in DMSO (*Table 1*). This observation suggests that the organic solvent may access the enzyme’s active site and interfere with the rates of individual reaction steps [23, 24].

**Operational stability of DestiTA in aqueousorganic solutions: effect of the T199Q substitution**

The operational stability of a biocatalyst is an important factor determining enzyme performance under specific reaction conditions. It is typically assessed by residual enzyme activity under reaction conditions; i.e., in the presence of substrates, excess PLP, and other additives [18]. The PLP excess fundamentally distinguishes this experiment from the kinetic stability analysis, which is typically conducted with the holoenzyme without excess PLP.

**Fig. 6:**
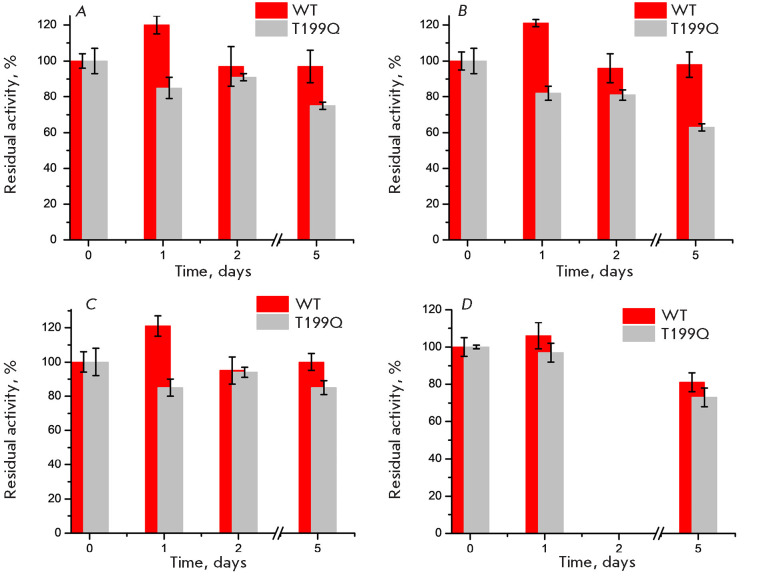
Residual activity of WT DestiTA (0.25 mg/mL) and T199Q incubated in 50 mM K-phosphate buffer (pH 8.0), containing 50 mM D-glutamate, 50 mM 3-methyl-2-oxobutyrate, 300 μM PLP, and supplemented with 10% (*v/v*) DMSO (*A*), 20% (*v/v*) DMSO (*B*), 10% (*v/v*) methanol (*C*), and in the absence of organic solvents (*D*). A value of 100% specific activity corresponds to 3.4 ± 0.2 U/mg for WT DestiTA and 2.8 ± 0.2 U/mg for T199Q

The operational stability of DestiTA is shown in *Fig. 6*. Notably, excess PLP effectively stabilizes the wild-type (WT) enzyme and, to a lesser extent, the T199Q variant. The effects of solvents on the holoenzyme dissociation rate constant are consistent with those previously observed (*Table 3*). However, the addition of free PLP does not compensate for the kinetic instability of the variant. In addition, excess PLP significantly stabilizes the active holoenzyme form, maintaining catalytic activity for several days. Under comparable conditions, but without excess PLP, the inactivation half-lives for the WT holoenzyme and T199Q are approximately 50 min and 7 min, respectively (*Table 3*).

Thus, the experimental results indicate that DestiTA stability decreases in the following solvent series: DMSO > methanol > Cyrene. Introduction of the T199Q substitution results in a slight increase in DestiTA thermal stability in both the buffer and water- organic solvent media and promotes holoenzyme stabilization in buffer. However, this effect is not retained under reaction conditions. Kinetic stability is a complex parameter that includes the stability of both the holoenzyme and apoenzyme, as well as the substrate-binding and cofactor-binding efficiencies. A relatively small (within one order of magnitude) increase in PLP-binding affinity is insufficient to reliably predict a change in the transaminase kinetic and operational stability.

## CONCLUSION

Despite the long history of studying PLP-dependent enzymes, the development of stable transaminase- based biocatalysts for industrial application remains a elusive in modern enzymatic catalysis. PLP binding in the active site of transaminases, in particular DestiTA, determines not only the enzyme’s catalytic properties but also its stability. Altering the PLP-binding site has a complex effect on a wide range of enzyme parameters; namely, catalytic efficiency, thermal stability, operational stability, and the stability in water-organic solvent solutions. In this context, DestiTA, as a moderately thermally stable enzyme, retains stability in buffers containing up to 20% of the organic solvent. The T199Q substitution enhances thermal stability and retention of enzyme activity in the reaction between D-alanine and α-ketoglutarate in water-organic solvent media, but it reduces its kinetic and operational stability. The introduction of the T199Q substitution stabilizes the holoenzyme but destabilizes the DestiTA apoenzyme and reduces the catalytic efficiency of the half-reaction of D-amino acid deamination. It can be concluded that increasing the stability of the protein globule (apoenzyme) is preferable for DestiTA stabilization in biotechnological processes, since holoenzyme stability can be achieved by increasing the PLP concentration in the reactor. The conducted studies further confirm the effectiveness of transaminase stabilization when there is an excess of free PLP.

## Abbreviations

DMSO: dimethyl sulfoxide
PLP: pyridoxal-5’-phosphate
PMP: pyridoxamine-5’-phosphate
TA: transaminase
DestiTA: transaminase from Desulfomonile tiedjei
LDH: lactate dehydrogenase
WT: wild type

## References

[1] Eliot AC., Kirsch JF. (2004). Pyridoxal phosphate enzymes: mechanistic, structural, and evolutionary considerations.. Annu Rev Biochem..

[2] Braunstein AE. (1973). Amino group transfer.. The Enzymes..

[3] Metzler DE., Ikawa M., Snell EE. (1954). A general mechanism for vitamin B6-catalyzed reactions.. J Am Chem Soc..

[4] Metzler CM., Metzler DE. (1987). Quantitative description of absorption spectra of a pyridoxal phosphate-dependent enzyme using lognormal distribution curves.. Anal Biochem..

[5] Braunstein AE., Shemyakin MM. (1953). Theory on amino acid metabolism processes catalyzed by pyridoxine enzymes.. Biokhimiia..

[6] Borisov VV., Borisova SN., Kachalova GS. (1978). Three-dimensional structure at 5 Å resolution of cytosolic aspartate transaminase from chicken heart.. J Mol Biol..

[7] Savile CK., Janey JM., Mundorff EC. (2010). Biocatalytic asymmetric synthesis of chiral amines from ketones applied to sitagliptin manufacture.. Science..

[8] Guo F., Berglund P. (2017). Transaminase biocatalysis: optimization and application.. Green Chem..

[9] Limanto J., Ashley ER., Yin J. (2014). A highly efficient asymmetric synthesis of vernakalant.. Org Lett..

[10] Slabu I., Galman JL., Lloyd RC., Turner NJ. (2017). Discovery, Engineering, and synthetic application of transaminase biocatalysts.. ACS Catal..

[11] Yi D., Bayer T., Badenhorst CPS. (2021). Recent trends in biocatalysis.. Chem Soc Rev..

[12] Desai AA. (2011). Sitagliptin manufacture: a compelling tale of green chemistry, process intensification, and industrial asymmetric catalysis.. Angew Chemie Int Ed Engl..

[13] O’Connell A., Haarr MB., Ryan J. (2025). Transaminase-triggered cascades for the synthesis and dynamic kinetic resolution of chiral N-heterocycles.. Angew Chemie Int Ed Engl..

[14] Ao YF., Pei S., Xiang C. (2023). Structure- and data-driven protein engineering of transaminases for improving activity and stereoselectivity.. Angew Chemie Int Ed Engl..

[15] Ferrandi EE., Monti D. (2017). Amine transaminases in chiral amines synthesis: recent advances and challenges.. World J Microbiol Biotechnol..

[16] Wu S., Snajdrova R., Moore JC., Baldenius K., Bornscheuer UT. (2021). Biocatalysis: Enzymatic synthesis for industrial applications.. Angew Chem Int Ed Engl..

[17] Roura Padrosa D., Alaux R., Smith P., Dreveny I., López-Gallego F., Paradisi F. (2019). Enhancing PLP-binding capacity of class-III ω-transaminase by single residue substitution.. Front Bioeng Biotechnol..

[18] Börner T., Rämisch S., Reddem ER. (2017). Explaining operational instability of amine transaminases: substrate-induced inactivation mechanism and influence of quaternary structure on enzyme–cofactor intermediate stability.. ACS Catal..

[19] Bakunova AK., Isaikina TY., Popov VO., Bezsudnova EY. (2022). Asymmetric synthesis of enantiomerically pure aliphatic and aromatic D-amino acids catalyzed by transaminase from Haliscomenobacter hydrossis.. Catalysts..

[20] Bakunova AK., Rudina IV., Popov VO., Bezsudnova EY. (2025). Contribution of second-shell residues to PLP-dependent transaminase catalysis: a case study of D-amino acid transaminase from Desulfomonile tiedjei.. Int J Mol Sci..

[21] Sherwood J., De bruyn M., Constantinou A. (2014). Dihydrolevoglucosenone (Cyrene) as a bio-based alternative for dipolar aprotic solvents.. Chem Commun (Camb)..

[22] Domínguez de María P. (2022). Biocatalysis and green solvents: trends, needs, and opportunities. In: Lozano P, ed. Biocatalysis in Green Solvents.. Elsevier;.

[23] Polizzi KM., Bommarius AS., Broering JM., Chaparro-Riggers JF. (2007). Stability of biocatalysts.. Curr Opin Chem Biol..

[24] Stepankova V., Bidmanova S., Koudelakova T., Prokop Z., Chaloupkova R., Damborsky J. (2013). Strategies for stabilization of enzymes in organic solvents.. ACS Catal..

